# “I Have No Clue What I Drunk Last Night” Using Smartphone Technology to Compare In-Vivo and Retrospective Self-Reports of Alcohol Consumption

**DOI:** 10.1371/journal.pone.0126209

**Published:** 2015-05-19

**Authors:** Rebecca Louise Monk, Derek Heim, Adam Qureshi, Alan Price

**Affiliations:** Department of Psychology, Edge Hill University, Ormskirk, Lancashire, United Kingdom; Oregon Health and Science University, UNITED STATES

## Abstract

**Aim:**

This research compared real-time measurements of alcohol consumption with retrospective accounts of alcohol consumption to examine possible discrepancies between, and contextual influences on, the different accounts.

**Method:**

Building on previous investigations, a specifically designed Smartphone technology was utilized to measure alcohol consumption and contextual influences in de facto real-time. Real-time data (a total of 10,560 data points relating to type and number of drinks and current social / environmental context) were compared with daily and weekly retrospective accounts of alcohol consumption.

**Results:**

Participants reported consuming more alcoholic drinks during real-time assessment than retrospectively. For daily accounts a higher number of drinks consumed in real-time was related to a higher discrepancy between real-time and retrospective accounts. This effect was found across all drink types but was not shaped by social and environmental contexts. Higher in-vivo alcohol consumption appeared to be related to a higher discrepancy in retrospectively reported weekly consumption for alcohol beverage types other than wine. When including contextual factors into the statistical models, being with two or more friends (as opposed to being alone) decreased the discrepancy between real-time and retrospective reports, whilst being in the pub (relative to being at home) was associated with greater discrepancies.

**Conclusions:**

Overall, retrospective accounts may underestimate the amount of actual, real-time alcohol consumed. Increased consumption may also exacerbate differences between real-time and retrospective accounts. Nonetheless, this is not a global effect as environmental and social contexts interact with the type of alcohol consumed and the time frame given for reporting (weekly vs. daily retrospective). A degree of caution therefore appears warranted with regards to the use of retrospective self-report methods of recording alcohol consumption. Whilst real-time sampling is unlikely to be completely error free, it may be better able to account for social and environmental influences on self-reported consumption.

## Introduction

Self-report measures are the bedrock of much research in the addictions [1], and it is generally accepted that this approach can be used as a reliable and valid method [2, 3]. By providing a systematic, structured and guided format for recording drinking, the time-line follow back interview (e.g., [4]), for example, is a widely used and highly regarded method for recording alcohol consumption by. Less structured approaches, on the other hand, ask that participants recall and record the total number of drinks consumed in a previous time period. However, the environments in which such retrospective assessments take place are often far removed from the settings in which the drinking occurs, by nature of their post hoc design [5]. Accordingly, the task of retrospective recall may encourage fabrication in an effort to satisfy the demands of the researchers [6,7]. Such a task may be particularly demanding cognitively as alcohol consumption can impair memory (c.f. [8, 9]) and because of the absence of environmental stimuli which may aid recall (c.f. [10]). Indeed, research suggests that variations in response to alcohol-related questions can be expected across contexts [11–13] and that both social and environmental factors are important determinants of a variety of alcohol-related cognitions and practices (c.f. for example,[14–17]). Resultantly, retrospective accounts of alcohol consumption may be expected to differ from those obtained in real world drinking contexts.

With the possible weaknesses of self-report measures in mind, in recent times there has been an increased focus on in-vivo assessment of alcohol consumption. The use of ecological momentary assessment (EMA), in particular, is thought to reduce the demands placed on autobiographical memory (c.f. [18–19]) and removes the potential for “parking-lot compliance” [20]. This approach also allows researchers to gather extensive data regarding respondents’ current behaviours and beliefs at different times over an extended period of assessment. The use of EMA to assess real-time substance use has been previously pioneered in the study of varying substance use, providing episode-based reports of real-time smoking and drinking [21–28]. This research has provided insights into the antecedents of real-time substance use, as well as demonstrating that in vivo accounts may differ from retrospective reports. This research has also paved the way for more systematic examinations of consumption patterns and impacts of contextual factors.

Building on this design, the purpose of this study is to examine and to compare possible variations between de facto real-time and retrospective recall of alcohol consumption. By using smartphone technology we hoped to gather instantaneous and rich data that are electronically time-stamped and help minimise reliance on retrospective accounts [29, 30]. We expected EMA using smartphone technology to be ‘context-aware’ [31] and to enable us to keep track of changes which may be particularly useful for monitoring behaviours which are episodic and bound contextually [17].

## Method

Following ethical approval from the University’s ethics boards (Edge Hill University Psychology Department Research Ethics Committee & Edge Hill University Faculty of Arts and Sciences Research Ethics Committee), participants were recruited though opportunity sampling on a university campus and through online adverts (intranet and social media). All participants gave fully informed written consent. This research complies with the World Medical Association Declaration of Helsinki.

### Design

A within participants design was utilised to investigate the difference between participants' in-vivo recorded alcohol consumption and their daily and weekly retrospective accounts of consumption. The environmental and social contexts in which participant responded and the type of alcohol consumed were also recorded.

### Participants

69 participants (18–36 years, M = 21.47, SD = 4.47) were recruited on a university campus. The majority of this sample was White British (95%) students (85.5%). 59% were male. Participants were offered monetary reimbursement (£7) or course credit by way of remuneration. Prior to further analyses, participants who failed to activate the application, or failed to complete at least 2 full response sessions on a single drinking occasion (*n* = 18) were removed from the data file. The data we collected on participant user experiences suggest that the majority did not find the app overly burdensome. Indeed, over 80% either agreed or strongly agreed that the App was convenient and easy to use, although there are clearly some time demands for taking part. We have no data on those who signed up but then never activated the application.

This decision was taken as these cases contributed no (or very little) data to the overall data set. Preliminary analyses revealed that there were no significant differences in demographic or baseline reports of alcohol consumption between these excluded participants and those who remained in the data set. Furthermore, the structured nature of the data meant that, despite this deletion, there were a substantial proportion of data points remaining for analytical purposes. Data regarding the number of each presented drink type, current social context and present environmental location were provided in each in-vivo hourly response session, meaning that participants provided 12 pieces of information about their drinking and current location every hour that they responded (12 data points). Given that participants completed an average of 4 sessions per drinking day, all participants provided an averaged minimum of 48 data pieces of information during the research. 22 participants provided responses for one day’s drinking (1056 data points), 16 provided responses for two drinking days (2158 data points), 8 provided responses for three drinking days (3158 points); and 4 provided responses for four days (4224 data points). In short, participants included within subsequent analyses provided 10, 560 data points during their in-vivo responding.

### Assessment Procedure

Following ethical approval, participants were recruited though opportunity sampling on a university campus and through online adverts (intranet and social media). All participants provided fully informed written consent. Those who signed up to take part attended a briefing session where they were instructed how to download and use the application. Participants' demographic details and information about their drinking (AUDIT) were also obtained by way of an electronically administered questionnaire.

Participants were asked to use the application to report as many separate drinking occasions as possible over the participation week. Once activated, the application triggered hourly participation prompts asking respondents to record their alcohol consumption in the last hour (see measure section for further detail). These hourly prompts continued until participants indicated that they had finished the dinking session.

The research team emailed participants 24 hours after the drinking session had finished and asked them to complete a short online survey in which they were asked to recall their previous alcohol consumption on the previous day. This process was repeated for every drinking occasion that the participants documented. At the end of the participation week participants provided their weekly retrospective reports electronically.

### Measures

Prior to taking part in the research, participants were asked standard questions about their age, gender, status on campus (student or non student). They were also asked questions about their drinking practices and related beliefs. These were assessed using two standardised questionnaires: First, the Alcohol Outcomes Expectancy Questionnaire (AOEQ, [32]), which is 34 item questionnaire (rated on a 6 point likert scale: 1 = no chance, and 6 = certain to happen) asking about the positive and negative outcomes that participants expect to result from drinking. A standardised, average of participants’ responses to positive items was calculated, giving a range of 1–6 (M = 4.13, SD =. 68). Second, the AUDIT was administered, which is a well established standardized measure of alcohol-related problems that is used in both clinical and non clinical settings. Its items assess 3 areas: harmful alcohol use, hazardous alcohol use and dependence symptoms and its raw score can be used to classify respondents based on their drinking. A score exceeding 8 (or 10 in some cases) is considered indicative of hazardous drinking [33]. The current participants’ mean AUDIT score was 9.19 (SD = 4.72).

The application itself asked participants a number of multiple choice questions which enquired about the respondents' current location (response options: Home, Work, Another’s Home, Restaurant, Bar-Pub-Club, Party, Sporting event, Other) and who they were with (response options: Alone, Colleague(s), Family/Partner, 1 friend, 2 or more friends, Other). The application presented numerous drink options. These options were labelled, pictorial representations of different drink types alongside a description of standard measurements (options: 1/2 pint beer/cider, 1 pint beer/cider, small bottle beer/cider, large bottle beer/cider, small glass wine, large glass wine, small spirit and mixed, large spirit and mixer, 1 short/shot, cocktail, other). Once ticked, participants could then record the number of that particular drink they had consumed. Using the same response format, follow up emails for the purposes of retrospective self-reports (24 hours and 1 week post hoc), also asked participants to record the type and quantity of alcohol consumed.

### Equipment

A Smartphone application was designed specifically for this research and it enabled participants to respond to questioning via the use of their own mobile phone. The application and interface were built using HTML and JavaScript and JavaScript's jQuery mobile library. Phone Gap was used to convert the web-based application into a native application that could be downloaded onto the users’ own device by scanning a QR code. Local Storage within the application was used to store temporarily all the users’ answers, before data were remotely transferred to Google Analytics. To facilitate data anonymity and allow researchers to track individual level data, individual participants' responses were tracked using a unique alias which was randomly generated by the application. The application was carefully designed and piloted to make the user interface as intuitive/user friendly as possible and there were no default answers set, in accordance with recommendations (c.f., [31]). Participants were instructed to activate the application when beginning a drinking session. It then provided hourly prompts that asked respondents to record what they were drinking. Hourly prompts elicited around 68% compliance rates (there is some slight ambiguity in this figure as some participants reported a signal issue which meant that they stated that they had responded but their data had not been captured on the server). Drinking cessation was assessed by the application. Specifically, the application required participants to indicate their immediate future drinking plans every time they responded. Here there were three options available to participants: Intend to continue drinking (in which case they were prompted again an hour later); Finish drinking for now but will continue later (in which case there was a 3 hour delay before the next prompt); Finish drinking for the day (in which case prompts ceased).

### Data Selection and Analytic strategy

Participants’ in-vivo records of the type and quantity of alcohol consumed were compared with their daily and weekly retrospective self-reports. In order to facilitate this, drinks of the same type that were consumed in different quantities were combined into broader categories. For example, ½ pint of beer or cider, 1 pint of beer or cider, and small or large bottles of beer or cider were combined into a single category (beer/cider). The same was done to create a further 3 categories: Wine (combining small and large glasses of wine), Spirits (combining single-25ml, double mixed-50ml-drinks and shots). Daily and weekly overall totals were also calculated for both in-vivo and retrospective drinking records. The average number of drinks consumed for these categories are summarized in Table 1.

**Table 1:** Participants’ averaged reported alcohol consumption across drink type and assessment time period.

	Daily Reports		Weekly Reports	
Drink Type	*In-vivo*	*Retro*	*In-vivo*	*Retro*
Beer/Cider	4.94 (5.17)	2.23 (2.49)	7.04 (5.56)	3.35 (3.35)
Wine	1.36(2.74)	.75 (1.55)	2.25 (4.19)	1.37 (2.54)
Spirits	1.53 (2.22)	.78 (1.47)	2.57 (4.22)	2.80 (4.95)
Other	.63 (1.52)	.41 (1.02)	1.25 (2.67)	1.31 (2.83)
Total Drinks	8.45 (5.97)	4.17 (3.02)	13.12 (8.83)	8.45 (6.85)

In-vivo reports were taken every hour on days where the application was activated by respondents. Analyses of daily consumption here therefore represent the total number of drinks that participants’ reported consuming (in real-time) over the course of their first day using the application. Day 1 of participation was selected in order that the maximal amount of data could be included within the daily in-vivo vs. retrospective analyses—not all participants (*n* = 22) took part for more than one day (i.e., recorded more than one drinking session). Nonetheless, weekly totals were calculated by summing the number of drinks recorded over every drinking occasion that was documented. This does mean that in some cases, participant’s real-time daily and weekly drinking totals were identical. However, these data are pertinent, as they still allow for both daily and weekly in-vivo vs. retrospective comparisons—shedding light on nature of self-report data. This was calculated by summing every hourly response. Participants’ daily retrospective accounts for that day (i.e., their self-reported consumption the day after) then provided the daily retrospective measure for analytical purposes. In the same vein, weekly totals were calculated by summing the number of drinks recorded across every drinking occasion that was documented in real-time. Participants’ weekly retrospective accounts at the end of the participation process (i.e., their self-reported consumption for the week) provided the weekly retrospective measure for analytical purposes.

Initial analyses indicated that there were significant differences between participants’ in-vivo and retrospective drinking accounts of the number of drinks consumed in both daily (*t* (50) *=* 5.99 *p* <. 001) and weekly assessments (*t* (50) = 3.39, *p* <.01) such that retrospective reports were lower than in-vivo accounts.

Four sets of models were analysed using multi-level mixed effects linear regressions (using xtmixed command in STATA 12 SE). A set of models was produced for each of the following criterion variables: Daily difference score, daily retrospective score, weekly difference score and weekly retrospective score. Unlike retrospective scores, for which models will show a direct relationship between recall consumption and the various predictors, the difference scores represent the discrepancy between real-time and recalled consumption. Higher difference scores indicate greater discrepancies between real-time and recalled consumption. Positive relationships with any predictors suggest that those predictors increase that discrepancy, whilst those predictors with negative relationships suggest that these variables reduce the discrepancy. Each model that was constructed followed the same analytical procedure: Model 1.) Overall in-vivo score Model 2.) Individual variations in drinking type—as it possible that people who are more likely to drink certain types of alcoholic beverages experience greater difficulty retrospectively reporting their drinking behavior. Model 3.) Individual variations in drinking type, social and environmental drinking locations and AUDIT scores. The final models are presented below—the other models are provided in the supporting information (S1 File).

## Results

Initial correlational analyses revealed that whilst daily in vivo reports were moderately correlated with daily retrospective reports (*r*. 52, *p* <.01), there was no significant correlation between weekly in vivo and retrospective reports (*r* =. 02, *p* >.05). All preliminary correctional analyses are reported in Table 2.

**Table 2:** Initial correlation analyses.

	Day 1 In-vivo Total	Day 1 Retro Total	Weekly In-vivo Total	Weekly Retro Total	Daily discrepancy score	Weekly discrepancy score
Day 1 In-vivo Total	1					
Day 1 Retro Total	0.5147**	1				
Weekly In-vivo Total	0.7464**	0.5266**	1			
Weekly Retro Total	0.1725	0.5582**	0.2317	1		
Daily discrepancy score	0.8631**	0.0112	0.5603**	-0.1277	1	
Weekly discrepancy score	0.5496**	0.084	0.736**	-0.4881**	0.5915**	1

* Correlation is significant at the 0.05 level (2-tailed)

** Correlation is significant at the 0.01 level (2-tailed).

### Daily difference score

The initial model showed that overall real-time alcohol consumption was significantly related to the daily difference score (Table A in S1 File), suggesting that higher alcohol consumption results in a higher discrepancy with recalled consumption. The individual variations in drinking type model (Table B in S1 File) showed the same pattern for all types of alcohol consumed (beer, wine, spirits and other). Table 3 shows that in the final model neither the context nor AUDIT variables had a significant relationship with daily difference score, while the individual variations in drinking type remained significant.

**Table 3:** Daily difference score predicted by In-vivo individual drink scores, context and AUDIT score; Wald Χ^2^ (12) = 231.29, p ≤ 0.01.

Factor	Coefficient	*P*	95% Confidence Intervals	
Day 1 In-vivo Beer	0.79	≤ 0.01	0.65	0.94
Day 1 In-vivo Wine	0.66	≤ 0.01	0.41	0.92
Day 1 In-vivo Spirits	0.67	≤ 0.05	0.07	1.28
Day 1 In-vivo Other	1.45	≤ 0.01	0.74	2.17
Day 1 Where (reference = home)				
Others home	-0.45	0.76	-3.29	2.39
Work	0.39	0.87	-3.81	4.49
Restaurant	3.95	0.13	-1.10	-9.01
Bar/Club/Pub	1.36	0.32	-1.31	4.03
Party	0.01	1.00	-5.96	5.98
Day 1 Who (reference = alone)				
Family	0.00	1.00	-3.64	3.65
2 + friends	-1.45	0.25	-3.90	1.00
AUDIT	0.11	0.23	-0.07	0.28
Constant	-3.20	≤ 0.01	-5.55	-0.86
Random-effects Parameters	Estimate	S.E.		
Standard deviation (residual)	2.04	0.22	1.64	2.52

### Recall after 1 day

Overall real-time alcohol consumption was significantly related to recalled alcohol consumption after 1 day (Table C in S1 File), suggesting a positive association between levels of real-time and recalled consumption. This was also found for the individual variations in drinking type model (Table D in S1 File) apart from for 'other' drinks. Table 4 shows that in the final model, neither the context nor AUDIT variables had a significant relationship with recalled alcohol consumption, while the 'beer' and 'wine' individual drink types remained significant.

**Table 4:** Recall after 1 day predicted by In-vivo individual drink scores, context and AUDIT score; Wald Χ^2^ (12) = 29.27, p ≤ 0.01.

Factor	Coefficient	*P*	95% Confidence Intervals	
Day 1 In-vivo Beer	0.21	≤ 0.01	0.06	0.35
Day 1 In-vivo Wine	0.34	≤ 0.05	0.08	0.59
Day 1 In-vivo Spirits	0.33	0.29	-0.28	0.93
Day 1 In-vivo Other	-0.45	0.21	-1.17	0.26
Day 1 Where (reference = home)				
Others home	0.45	0.76	-2.39	3.29
Work	-0.34	0.87	-4.49	3.81
Restaurant	-3.95	0.13	-9.01	1.10
Bar/Club/Pub	-1.35	0.32	-4.03	1.31
Party	-0.01	1.00	-5.98	5.96
Day 1 Who (reference = alone)				
Family	0.00	1.00	-3.64	3.64
2 + friends	1.45	0.25	-0.99	3.90
AUDIT	-0.11	0.23	-0.28	0.07
Constant	3.20	≤ 0.01	0.87	5.55
Random effects Parameters	Estimate	S.E.		
Standard deviation (residual)	2.04	0.22	1.64	2.52

### Weekly difference score

The initial model showed that overall real-time alcohol consumption was significantly related to the weekly difference score (Table E in S1 File), suggesting that higher alcohol consumption results in a higher discrepancy with recalled consumption. The individual variations in drinking type model (Table F in S1 File) showed the same pattern (except for ‘wine’ and 'other' drinks). Table 5 shows that for the final model, the individual variations in drinking type remained the same as in the prior model. AUDIT score was not significantly related to the weekly difference score. For the 'where' context variable (environmental context), both being at work or at a bar, club or pub was associated with a significantly greater discrepancy in real-time and recalled weekly consumption, compared to being at home. For the 'who' context variable (social context), being with family or with two or more friends was associated with significantly lower discrepancy in real-time and retrospectively recalled consumption, compared to being alone.

**Table 5:** Weekly difference score predicted by In-vivo individual drink scores, context and AUDIT score; Wald Χ^2^ (12) = 23.74, p = ≤ 0.05.

Factor	Coefficient	*P*	95% Confidence Intervals	
Weekly In-vivo Beer	0.61	≤ 0.01	0.26	0.97
Weekly In-vivo Wine	1.10	≤ 0.06	0.63	1.57
Weekly In-vivo Spirits	1.20	≤ 0.01	0.36	2.04
Weekly In-vivo Other	0.58	0.17	-0.24	1.40
Day 1 Where (reference = home)				
Others home	6.42	0.09	-1.02	13.85
Work	14.43	≤ 0.01	3.82	25.04
Restaurant	-2.94	0.66	-16.16	10.27
Bar/Club/Pub	10.78	≤ 0.01	3.64	17.91
Party	9.56	0.22	-5.84	24.96
Day 1 Who (reference = alone)				
Family	-10.62	≤ 0.05	-20.20	-1.05
2 + friends	-6.25	≤ 0.05	-12.30	-0.21
AUDIT	-0.10	0.66	-0.57	0.36
Constant	-7.97	≤ 0.01	-13.94	-2.00
Random effects Parameters	Estimate	S.E.		
Standard deviation (residual)	5.37	0.59	4.34	6.65

### Recall after 1 week

Neither overall consumption or individual variations in drinking type (Table G & Table H in S1 File) were significantly related to recalled consumption after one week. Table 6 shows that of the individual variations in drinking type, beer consumption is now significantly related to recalled alcohol consumption after one week. AUDIT score was not significantly related to recalled alcohol consumption after one week. For the 'where' context variable (environmental context), as compared to being at home, both being at work and being at a bar, club or pub were negatively associated with recalled alcohol consumption. This perhaps suggests that being in these social contexts impairs later recall of the amount of alcohol consumed. For the 'who' context variable (social context), both being with family and being with two or more friends were positively associated with recalled alcohol consumption, as compared to being alone.

**Table 6:** Recall after 1 week predicted by In-vivo individual drink scores, context and AUDIT score; Wald Χ^2^ (12) = 23.74, p = ≤ 0.05.

Factor	Coefficient	*P*	95% Confidence Intervals	
Weekly In-vivo Beer	0.39	≤ 0.05	0.03	0.74
Weekly In-vivo Wine	-0.10	0.68	-0.57	0.37
Weekly In-vivo Spirits	-0.20	0.65	-1.03	0.64
Weekly In-vivo Other	0.42	0.31	-0.40	1.24
Day 1 Where (reference = home)				
Others home	-6.42	0.09	-13.85	1.02
Work	-14.42	≤ 0.01	-25.04	-3.82
Restaurant	2.94	0.66	-10.27	16.16
Bar/Club/Pub	-10.78	≤ 0.01	-17.91	-3.64
Party	-9.57	0.22	-24.96	5.84
Day 1 Who (reference = alone)				
Family	10.62	≤ 0.05	1.05	20.20
2 + friends	6.25	≤ 0.05	0.21	12.30
AUDIT	0.10	0.66	-0.36	0.57
Constant	7.97	≤ 0.01	2.00	13.94
Random effects Parameters	Estimate	S.E.		
Standard deviation (residual)	5.37	0.59	4.34	6.65

### Model comparisons

Likelihood ratio tests were used to compare the models. For both the daily difference score and recall after one day models, there was a marginally significant difference between the overall in-vivo score model compared to the individual drink in-vivo scores model (*p* = 0.09). The model adding context information and AUDIT scores was not significantly different from either the overall in-vivo score or individual in-vivo drink score models (both *p*'s > 0.05). For both the weekly difference score and weekly recall models, there was no significant difference between the overall and individual in-vivo models (*p* > 0.05), but the model with context and AUDIT score was significantly different to the individual in-vivo model (*p* < 0.05) and marginally so when compared to the overall in-vivo model (*p* = 0.08).

## Discussion

Overall the results from this research suggest that both daily and weekly retrospective reports regarding the quantities of alcohol consumed are not in-line with records provided in real-time (in-vivo). Specifically, participants appear to retrospectively under-report the number of drinks that they reportedly consumed in real-time. Although not discussed in this paper, this was further supported by qualitative reports which declared, for example, “I have no idea what I drunk last night”. Examining these findings in more detail reveals a complex picture of alcohol consumption reporting. Specifically, alcohol consumption reports may be impacted by the time-delay between drinking and recall, the quantity and type of drink consumed, and the social and environmental contexts in which drinking occurred.

Analyses of accounts of the previous day’s consumption revealed that increases in real-time drinking were associated with increases in the number of retrospectively recalled drinks. However, difference score analyses indicated that higher numbers of drinks consumed in real-time were related with a higher discrepancy between real-time and retrospective accounts, across almost all types of drink (the only exception being for “other” drinks). Social and environmental contexts, on the other hand, were not significant predictors of daily retrospective reports or difference scores.

For weekly accounts, higher in-vivo alcohol consumption also appeared to be related to a higher discrepancy in retrospectively reported consumption. It is certainly the case that arithmetically there is more 'room' for discrepancy when the quantities are larger. However, our findings raise the possibility that traditional retrospective report techniques may underestimate the consumption of heavy drinkers. On the other hand, when examining individual drink types this pattern was not evident for wine. As greater effortful processing is associated with better task performance [34], the heightened difficulty of recalling weekly as opposed to daily drinking [35] may therefore be an important determinant of performance. Items which are more unusual within memory have been postulated to have an attentional draw [36], meaning that they elicit more detailed encoding for the purpose of long term memory [37]. In the case of the current findings, drinks which were less frequently recorded within this cohort (e.g., wine) may therefore be recalled more accurately because of their relative salience. Conversely, more frequently consumed drinks (e.g., beer/cider and spirits) may be less salient, thus they be more poorly recalled and under-reported.

There was also a variation between the findings of daily and weekly retrospective models when the effects of environmental contexts were included. Whilst these contexts were not significant predictors of daily retrospective reports (or difference scores), their inclusion had a significant predictive value for weekly retrospective reports and difference scores. Here, alcohol consumption across individual drinks ceased to be significant predictors in the model. However, being with two or more friends (as opposed to being alone) became a significant predictor that was associated with an increase in retrospective reports and with a decrease in the discrepancy between real-time and retrospective reports. In this vein, it is possible that in the time preceding the final weekly self-report, respondents had time to share and exchange accounts (with their friends) of their previously shared drinking experiences. This sharing of past experiences is a highly valued part of the social drinking experiences [38, 39] and may, in turn, have contributed to the observed increase in retrospective reporting, and the corresponding decrease in the discrepancies between real-time accounts. This is clearly conjecture at this stage, and further research is required to test this hypothesis. Nonetheless, this process would also mirror the well-researched phenomenon of social reconstruction and memory conformity in forensic psychology (e.g., [40]) which indicates that those who share in an experience or witness an event will socially (re) construct their memories/accounts of an event.

It is also interesting to note that whilst there was no significant interaction between social and environmental contexts, the latter was a significant predictor of weekly retrospective reports. Here, being in the pub (relative to being at home) was associated with a decrease in retrospective reports and a heightened difference score. In other words, the pub environment seemed to increase the discrepancy between real-time reports of consumption and weekly retrospective recall consumption. One explanation for this finding may be offered by alcohol myopia theory [41] which suggests that contextual variations in attention impede later recall. These findings are also inline with previous research indicating a contextual variation in alcohol-related cognitions [12, 13, 42–44]. However, the fact that this contextual impact was not evident in daily reports suggests that this context-related narrowing of perceptual and cognitive functioning may be exacerbated over longer time frames, possibly as memory traces begin to fade.

An alternative explanation of the discrepancies between retrospective and real time recall may lie in differences in consumption as a function of contexts. From this perspective, contextual variations in reporting could be explained by variations in levels of alcohol-induced cognitive impairments—by which certain contexts are associated with more drinking (and elevated memory impairment). However, preliminarily analyses indicated that there was no significant difference in the total quantities of drinks recorded in real-time between the home and pub contexts. These findings may reflect 'pre- drinking / loading' practices (c.f., [45]) in student samples. In line with myopia theory, the larger discrepancies between real time and retrospective reporting in pub relative to home contexts may therefore be attributable to impaired self-monitoring in environments with a high salience of alcohol-related stimuli. In view of research indicating that recall is aided in contexts where encoding occurs [10], it is also possible that discrepancies arose because alcohol consumption in pubs was recalled in the home environment.

Further research in regard to this discrepancy between weekly and daily retrospective accounts is strongly recommended, as well as further research to support the development of real-time data collection methods. Extending this research to examine longer time recording frames may also prove beneficial, as would further analyses comparing real-time accounts with retrospective reports elicited by the 'gold standard' time-line follow back interview (c.f. [4]). It must also be acknowledged that, despite the high level of anonymity in the current research, real-time reports may also be prone to biases owing to demand characteristics [6]. It is asserted that judgment anticipation may increase the desire to self-present in a particular manner dependent on the context [34]. Real-time as opposed to retrospective reporting contexts may therefore result in differing levels of self presentation concern. For instance, recording the total number of drinks consumed over a past night/week may heighten the perception of potentially negative judgements/assessments from others—as these numbers are likely to be higher than the number of drinks consumed in a previous hour. In other words, declaring that one has had two drinks is unlikely to elicit as strongly negative response but proclaiming that one has consumed 10 drinks may have negative connotations. The psychological need to self-present may therefore be postulated to be stronger in retrospective reports. Nonetheless, this does not preclude the potential for reporting bias in real-time reports and this must therefore be acknowledged as a potential issue in the current research. Finally, access to a mobile phone was a pre-requisite for taking part, as mobile devices were not supplied. It was considered prudent to maximise the usability of the application and we believed that participant’s familiarity with their own phone’s features and operating system would maximise their interaction with the application. Given that the majority of students now have mobile devices, this was not deemed a large issue for generalisability however it should be noted that the present results can, of course, not be reliably believed to generalise to students without access to such technology.

It is also feasible that participants may have had difficulties in monitoring which drinks they had previously recorded—a telescoping effect. This leaves the possibility that some “double counting” of drinks may have occurred, although it is hoped that the hourly timing intervals will have limited the severity of this effect [46]. Finally, it should be noted that although these data were recorded in real-time, this does not preclude the possibility that participants forgot to record every single drink. Similarly, we cannot exclude the prospect that participants consumed alcohol on days when they did not use the application, meaning that these data will effectively have been missed. Ann additional degree of caution needs to be exercised when assessing the relative quantities of drinking reported by the two methods as drinking occasions not recorded in real-time were not included in the dataset and it is feasible that they could have been included in retroactive recall sessions. That said, given that people recalled less drinking retrospectively than in-vivo, it is indeed possible that retrospective reports under-report alcohol consumption to a greater degree than recorded in this study. The use of both the application and follow up assessment on every week day (drinking or not) may therefore provide greater data in this regard. The additional use of objective measures of consumption, such as a breathalysers or Secure Continuous Remote Alcohol Monitors (SCRAM), alongside in-vivo self-monitoring, is therefore recommended. This would also allow for an examination of how intoxication moderates the impact of retrospective recall, which cannot be assessed presently. An expansion of the current research to increase the current sample size would also be recommended, although it is believed that some of the potential concern in this regard has been ameliorated by the large number of data points supporting the current statistical analyses, and the relatively strong effect sizes revealed. Utilising a wider number of non students would also be advisable as differences in the student drinking culture [47] may mean that results cannot be generalised to the wider population. Finally, only four participants used the application for four days. In order to assess longer drinking periods, it may therefore be advisable for future research to increase participant recompense with a view to motivating respondents to extend their use of the application. Future research could also examine which factors motivate extended use of the application. It is possible that some participants may, for example, have used the application to monitor their drinking in an effort to reduce their consumption.

Overall the general discrepancy between in-vivo and retrospective accounts of consumption suggests that the use of self-report measures requires careful consideration within alcohol research. Compared with that the introduction of novel and cost effective ways of measuring alcohol consumption appears promising. This is not to say that real-time sampling is free from error, however, it may offer researchers a methodological tool which, in conjunction with others, may allow for greater insight into alcohol consumption in different environments. It therefore constitutes a further step towards developing research methods that may prove to be more valid than retrospective self-report measures.

### Declaration

The authors declare no conflicts of interest. This material has not been published in whole or in part elsewhere, not is the paper being currently being considered for publication elsewhere. All authors have been personally and actively involved in substantive work leading to the report, and will hold themselves jointly and individually responsible for its content. All relevant ethical safeguards have been met in relation to patient or subject protection, or animal experimentation, including, with a full review by an appropriate ethical review committee. This research complies with the World Medical Association Declaration of Helsinki.

## Supporting Information

S1 File This file contains multiple tables (A-H), whose individual captions are contained within. (DOC)
