# Supplementary material for: “I Have No Clue What I Drunk Last Night” Using Smartphone Technology to Compare In-Vivo and Retrospective Self-Reports of Alcohol Consumption
Source: PLoS One. 2015 May 19;10(5):e0126209. doi: 10.1371/journal.pone.0126209 (PMC4437777; doi:10.1371/journal.pone.0126209)
Supplement: S1 File — (DOC) [file pone.0126209.s001.doc]

Table A. Daily difference score predicted by In-vivo total score; Wald Χ^2^ (1) = 148.95, p ≤ 0.01

| Factor | Coefficient | *P* | 95% Confidence Intervals | |
| --- | --- | --- | --- | --- |
| Day 1 In-vivo Total | 0.74 | ≤ 0.01 | 0.62 | 0.86 |
| Constant | -1.96 | ≤ 0.01 | -3.19 | -0.73 |

| Random-effects Parameters | Estimate | Standard Error | 95% Confidence Intervals | |
| --- | --- | --- | --- | --- |
| Standard deviation (residual) | 2.56 | 0.25 | 2.11 | 3.11 |

Table B. Daily difference score predicted by In-vivo individual drink scores; Wald Χ^2^ (4) = 159.21, p ≤ 0.01

| Factor | Coefficient | *P* | 95% Confidence Intervals | |
| --- | --- | --- | --- | --- |
| Day 1 In-vivo Beer | 0.79 | ≤ 0.01 | 0.65 | 0.93 |
| Day 1 In-vivo Wine | 0.65 | ≤ 0.01 | 0.39 | 0.91 |
| Day 1 In-vivo Spirits | 0.54 | ≤ 0.01 | 0.20 | 0.89 |
| Day 1 In-vivo Other | 0.93 | ≤ 0.01 | 0.43 | 1.43 |
| Constant | -1.96 | ≤ 0.01 | -3.19 | -0.73 |

| Random-effects Parameters | Estimate | Standard Error | 95% Confidence Intervals | |
| --- | --- | --- | --- | --- |
| Standard deviation (residual) | 2.49 | 0.25 | 2.06 | 3.03 |

*Table C. Recall after 1 day predicted by In-vivo total score; Wald Χ^2^ (1) = 18.38, p ≤ 0.01*

| Factor | Coefficient | *P* | 95% Confidence Intervals | |
| --- | --- | --- | --- | --- |
| Day 1 In-vivo Total | 0.26 | ≤ 0.01 | 0.14 | 0.38 |
| Constant | 1.96 | ≤ 0.01 | 0.73 | 3.19 |

| Random-effects Parameters | Estimate | Standard Error | 95% Confidence Intervals | |
| --- | --- | --- | --- | --- |
| Standard deviation (residual) | 2.56 | 0.25 | 2.11 | 3.11 |

Table D. Recall after 1 day predicted by In-vivo individual drink scores; Wald Χ^2^ (4) = 21.94, p ≤ 0.01

| Factor | Coefficient | *P* | 95% Confidence Intervals | |
| --- | --- | --- | --- | --- |
| Day 1 In-vivo Beer | 0.21 | ≤ 0.01 | 0.07 | 0.35 |
| Day 1 In-vivo Wine | 0.35 | ≤ 0.01 | 0.09 | 0.61 |
| Day 1 In-vivo Spirits | 0.46 | ≤ 0.01 | 0.11 | 0.80 |
| Day 1 In-vivo Other | 0.07 | 0.79 | -0.43 | 0.57 |
| Constant | 1.90 | ≤ 0.01 | 0.70 | 3.10 |

| Random-effects Parameters | Estimate | Standard Error | 95% Confidence Intervals | |
| --- | --- | --- | --- | --- |
| Standard deviation (residual) | 2.50 | 0.25 | 2.06 | 3.03 |

*Table E. Weekly difference score predicted by In-vivo total score; Wald Χ^2^ (1) = 60.28, p ≤ 0.01*

| Factor | Coefficient | *P* | 95% Confidence Intervals | |
| --- | --- | --- | --- | --- |
| Weekly In-vivo Total | 0.82 | ≤ 0.01 | 0.61 | 1.03 |
| Constant | -6.09 | ≤ 0.01 | -9.36 | -2.83 |

| Random-effects Parameters | Estimate | Standard Error | 95% Confidence Intervals | |
| --- | --- | --- | --- | --- |
| Standard deviation (residual) | 6.60 | 0.65 | 5.43 | 8.01 |

Table F. Weekly difference score predicted by In-vivo individual drink scores; Wald Χ^2^ (4) = 10.03, p ≤ 0.05

| Factor | Coefficient | *P* | 95% Confidence Intervals | |
| --- | --- | --- | --- | --- |
| Weekly In-vivo Beer | 0.73 | ≤ 0.01 | 0.42 | 1.03 |
| Weekly In-vivo Wine | 1.24 | ≤ 0.06 | 0.80 | 1.68 |
| Weekly In-vivo Spirits | 0.67 | ≤ 0.01 | 0.23 | 1.10 |
| Weekly In-vivo Other | 0.39 | 0.25 | -0.27 | 1.06 |
| Constant | -5.43 | ≤ 0.01 | -8.63 | 2.23 |

| Random-effects Parameters | Estimate | Standard Error | 95% Confidence Intervals | |
| --- | --- | --- | --- | --- |
| Standard deviation (residual) | 6.20 | 0.61 | 5.10 | 7.52 |

Table G. Recall after 1 week predicted by In-vivo total score; Wald Χ^2^ (1) = 2.89, p = 0.09

| Factor | Coefficient | *P* | 95% Confidence Intervals | |
| --- | --- | --- | --- | --- |
| Weekly In-vivo Total | 0.18 | 0.09 | -0.03 | 0.39 |
| Constant | 6.09 | ≤ 0.01 | 2.83 | 9.36 |

| Random-effects Parameters | Estimate | Standard Error | 95% Confidence Intervals | |
| --- | --- | --- | --- | --- |
| Standard deviation (residual) | 6.60 | 0.65 | 5.43 | 8.01 |

Table H. Recall after 1 week predicted by In-vivo individual drink scores; Wald Χ^2^ (4) = 10.03, p ≤ 0.05

| Factor | Coefficient | *P* | 95% Confidence Intervals | |
| --- | --- | --- | --- | --- |
| Weekly In-vivo Beer | 0.27 | 0.08 | -0.03 | 0.58 |
| Weekly In-vivo Wine | -0.24 | 0.29 | -0.68 | 0.20 |
| Weekly In-vivo Spirits | 0.33 | 0.13 | -0.10 | 0.77 |
| Weekly In-vivo Other | 0.61 | 0.07 | -0.06 | 1.27 |
| Constant | 5.43 | ≤ 0.01 | 2.22 | 8.63 |

| Random-effects Parameters | Estimate | Standard Error | 95% Confidence Intervals | |
| --- | --- | --- | --- | --- |
| Standard deviation (residual) | 6.20 | 0.61 | 5.10 | 7.52 |
